# Comparative Analysis of Unsupervised Protein Similarity Prediction Based on Graph Embedding

**DOI:** 10.3389/fgene.2021.744334

**Published:** 2021-09-22

**Authors:** Yuanyuan Zhang, Ziqi Wang, Shudong Wang, Junliang Shang

**Affiliations:** ^1^School of Information and Control Engineering, Qingdao University of Technology, Qingdao, China; ^2^College of Computer Science and Technology, China University of Petroleum (East China), Qingdao, China; ^3^School of Information Science and Engineering, Qufu Normal University, Rizhao, China

**Keywords:** protein similarity, graph embedding, gene ontology, link prediction, DTW algorithm

## Abstract

The study of protein–protein interaction and the determination of protein functions are important parts of proteomics. Computational methods are used to study the similarity between proteins based on Gene Ontology (GO) to explore their functions and possible interactions. GO is a series of standardized terms that describe gene products from molecular functions, biological processes, and cell components. Previous studies on assessing the similarity of GO terms were primarily based on Information Content (IC) between GO terms to measure the similarity of proteins. However, these methods tend to ignore the structural information between GO terms. Therefore, considering the structural information of GO terms, we systematically analyze the performance of the GO graph and GO Annotation (GOA) graph in calculating the similarity of proteins using different graph embedding methods. When applied to the actual Human and Yeast datasets, the feature vectors of GO terms and proteins are learned based on different graph embedding methods. To measure the similarity of the proteins annotated by different GO numbers, we used Dynamic Time Warping (DTW) and cosine to calculate protein similarity in GO graph and GOA graph, respectively. Link prediction experiments were then performed to evaluate the reliability of protein similarity networks constructed by different methods. It is shown that graph embedding methods have obvious advantages over the traditional IC-based methods. We found that random walk graph embedding methods, in particular, showed excellent performance in calculating the similarity of proteins. By comparing link prediction experiment results from GO(DTW) and GOA(cosine) methods, it is shown that GO(DTW) features provide highly effective information for analyzing the similarity among proteins.

## Introduction

Proteomics essentially refers to the study of the characteristics of proteins on a large scale, including the expression level of proteins, the functions of proteins, protein–protein interactions, and so forth. The study of proteome not only provides the material basis for the law of life activities but can also provide the theoretical basis and solutions for elucidating and solving the mechanism of many diseases (Xi et al., 2020a). However, at present, research on the function of proteins is lacking. The functions of proteins encoded by most of the newly discovered genes by genome sequencing are unknown. For those whose functions are known, their functions have mostly been inferred by methods such as homologous gene function analogy. Therefore, using computational methods to explore the similarity between proteins can effectively improve the efficiency of proteomic studies.

Gene Ontology (GO) (Harris, 2004) describes the function of genes It is a standardized description of the characteristics of genes and gene products, enabling bioinformatics researchers to uniformly summarize, process, interpret, and share the data of genes and gene products. It provides the representation of biological knowledge through structured and controlled terms. GO includes three kinds of ontologies: Biological Processes (BPs), Cell Components (CCs), and Molecular Functions (MFs). The words in the three kinds of ontologies are related to each other and form a Directed Acyclic Graph (DAG), wherein a node denotes a GO term, while an edge denotes a kind of relationship between two GO terms. Therefore, it is of great significance to study the similarity of proteins based on the graph characteristics of GO to explore the function of proteins.

GO has been widely studied in the field of biology (Xi et al., 2020b). GO terms have been used to annotate many biomedical databases [e.g., UniProt database (UniProt Consortium, 2015) and SwissProt database (Amos and Brigitte, 1999)]. The characteristics and structure of GO have made GO terms the basis of functional comparison between gene products (Pesaranghader et al., 2014). GO annotation defines the semantic similarity of genes (proteins) and provides a basis for measuring the functional similarity of proteins. The more information two GO terms share, the more similar they are, and the more the similarity between the proteins annotated by the two GO terms (Hu et al., 2021). In earlier studies, many researchers analyzed protein–protein interaction (PPI) based on GO (Sevilla et al., 2005). Studies on computing protein similarity using GO mainly focus on the IC of GO terms, which is widely used to identify relations between proteins. The uniqueness of GO terms is often evaluated by taking the average of the IC of two terms. The IC of a term depends on the annotating corpus (Sevilla et al., 2005). Three IC-based methods—Resnik’s (Resnik, 1999), Rel’s (Paul and Meeta, 2008), and Jiang and Conrath’s (Jiang and Conrath, 1997)—have been introduced from natural language taxonomies by Lord et al. (2003) to compare genes (proteins). Although the abovementioned methods are used to calculate semantic similarity between two GO terms to achieve good results, they only consider the amount of information of common nodes. They do not consider the information differences between the nodes themselves and ignore the structural information of the terms. The result of term comparison is a rough estimate. For example, in Resnik’s method, if the ancestors of two terms are the same, then the similarity of two terms in any layer is not different and cannot be compared. Obviously, this is unreasonable.

This study merged the three categories of ontologies and GO annotations into a large graph called the GO Annotation (GOA) graph. We used three categories of ontologies transformed into a GO graph. Effective graph analysis on GOA and GO graphs can improve our understanding of the structure and node information of GO and proteins. Using the GOA information of the proteins, the similarity among proteins can be calculated, and the relationship between proteins can be predicted. In recent years, graph learning-based analytical methods have made remarkable progress in bioinformatics and other fields (Xi et al., 2021). At present, graph learning-based analytical methods focuses on dynamic graphs. Methods such as SDNE (Wang et al., 2016), DeepWalk (Perozzi et al., 2014), LINE (Tang et al., 2015), Node2vec (Grover and Leskovec, 2016), and SINE (Wang et al., 2020) have been widely used for unsupervised feature learning in the field of data mining and natural language processing. The edge prediction task is applied to the PPI prediction to find new protein interaction relationships. They also provide a basis for calculating protein similarity based on GO, such as GO2vec (Zhong et al., 2019), which used the Node2vec algorithm to compute the functional similarity between proteins.

**FIGURE 1:**
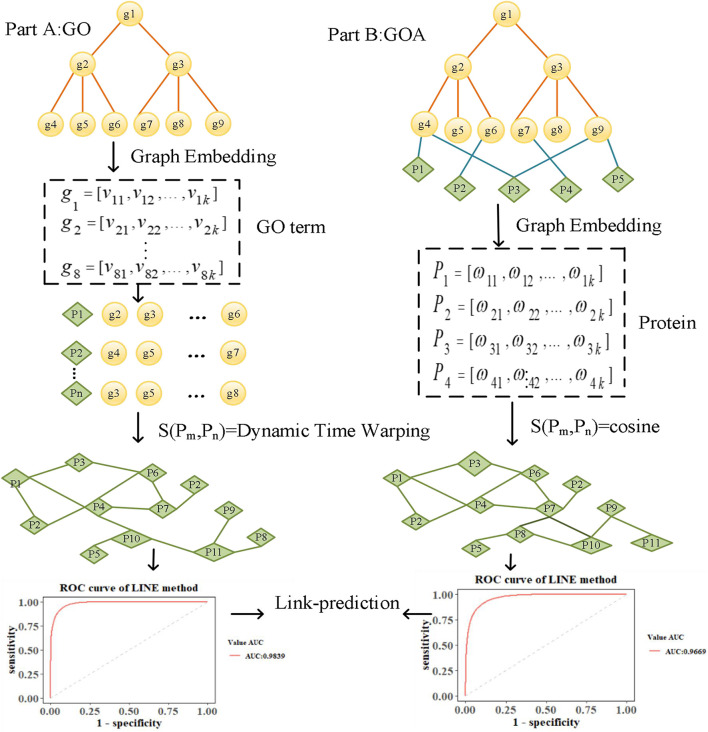
Framework for analyzing protein similarity.

To explore the performance of graph embedding methods in measuring protein similarity based on GO and GOA, we used four typical graph embedding methods to learn the features of GO terms and proteins. These methods can be divided into two categories. The first category is the random walk method, such as the DeepWalk and Node2Vec methods. The DeepWalk method uses the truncated random walk strategy to obtain the sequence of nodes and point embedding obtained from learning with Word2Vec (Goldberg and Levy, 2014). Node2Vec uses biased random walk to generate a node sequence by balancing the Breadth First Search (BFS) and Depth First Search (DFS) of the graph. The second category is based on deep learning, such as SDNE and LINE methods. SDNE uses an auto-encoder to optimize the first-order and second-order similarity simultaneously, while LINE optimizes the orders of similarity separately. As a result, their learned node embedding can retain the local and global graph structure and is robust to sparse networks. We introduce the overall flowchart of this paper in Figure 1, which is divided into two parts. Firstly, in Part A, the features of GO terms are learned based on the GO graph using graph embedding methods. The similarity of proteins is then calculated based on the features of their annotated GO terms by Dynamic Time Warping (DTW) distance (Lou et al., 2016). Secondly, in Part B, the features of proteins are learned based on the GOA graph directly. Then, the cosine similarity of the corresponding features is calculated to measure the similarity of protein. Finally, a link prediction (Li et al., 2018) experiment is performed in the screened-out protein similarity networks, using the area under the curve (AUC) (Lobo, 2010) and area under the precision-recall curve (AUCPR) (Yu and Park, 2014) to evaluate the reliability of the protein network constructed by learned vectors.

## Materials and Methods

### Data Source and Preprocessing

We downloaded GO data in Open Biomedical Ontologies (OBO) format from the GO Consortium Website^1^. The GO protein annotations were obtained from the UniProt GOA website^2^. The Yeast dataset contained 2,887 proteins, and the Human dataset contained 9,677 proteins. The GO data were then preprocessed based on the following processes. First, since several GO terms annotate a protein, term–term relations of GO terms and term–protein annotations between GO terms and proteins were combined into a GOA graph. Second, the GO terms were then transformed into an undirected, unweighted GO graph, regardless of the type and direction of the relationship. We summarize the numbers of GO terms and edges in Table 1.

**TABLE 1:** Characteristics of GO graphs.

Gene ontology	Term	Edges
BP*	30,705	71,530
CC**	4,380	7,523
MF***	12,127	13,658

*Biological Processes, **Cell Components, and ***Molecular Functions.

### Method

Based on different graph embedding methods, the feature of GO terms and proteins was learned into vector representations by fusing GO and GOA graph topologies, respectively. Thus, we could capture the global information based on the graph embedding method, and its learned vectors could calculate the similarity between proteins by the DTW distance and cosine similarity.

#### Introduction of Different Graph Embedding Methods

**FIGURE 2:**
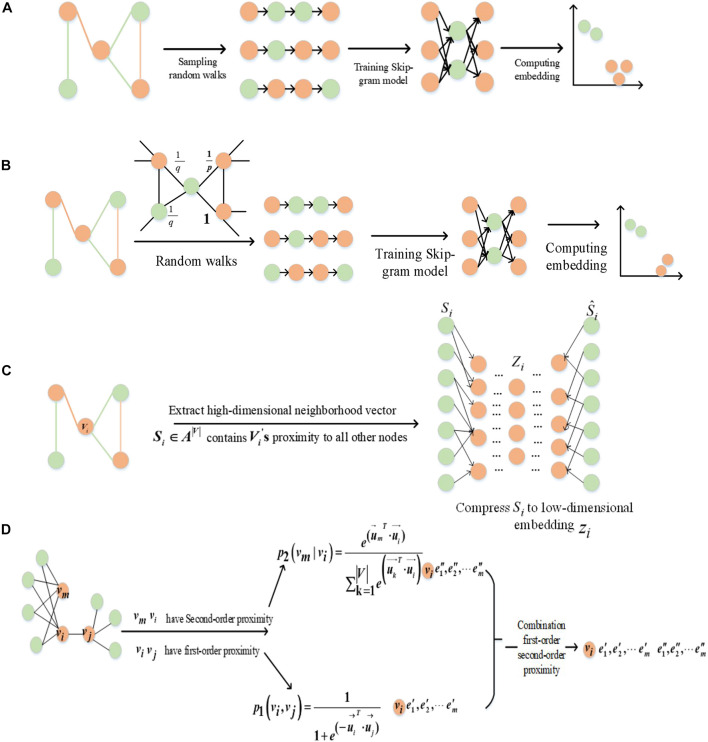
Framework for graph embedding method. **(A)** DeepWalk, **(B)** Node2vec, **(C)** SDNE, and **(D)** LINE.

In this paper, we used the methods of graph embedding based on random walk and deep learning to learn the features of GO terms and proteins through fusing the topology of GO and GOA graphs, respectively. Random walk-based methods include DeepWalk (Perozzi et al., 2014) and Node2vec (Grover and Leskovec, 2016). The DeepWalk method is divided into two parts: random walk to obtain node sequences and to generate node embedding. Random walk is used to obtain the local information of the node in the graph, and the embedding reflects the local structure of the node in the graph. The path length is controlled by setting the parameter walk-length (*_*L*_*). The more neighborhood nodes (higher-order neighborhood nodes) two nodes have, the more similar they are. Figure 2A illustrates the DeepWalk algorithm flow. Node2vec method sets two hyper-parameters *p* and *q* to control the random walk and adopts a flexible biased random walk procedure that smoothly combines BFS and DFS to generate node sequences. Figure 2B illustrates the Node2vec algorithm flow. Nodes *c*_*i*_ are generated based on the following distribution:


(1) $$P\left(c_{i}=x|c_{i-1}=t\right)=\left\{ \begin{array}{c} \frac{\pi_{tx}}{Z}\;\left(if\left(t,x\right)\in E\right)\\ 0\;\left(otherwise\right) \end{array}\right.$$

where π_*tx*_ is the transition probability between nodes *t* and *x*, and *Z* is the normalization constant. According to the node context information, node sequences are generated by setting the sizes of the hyper-parameters *p* and *q* to control the random walk strategy. The Skip-gram model is used to obtain the vector representation of the nodes. The random walk graph embedding of nodes reflects the local and global topology information of nodes in the graph.

The second kind of embedding method is SDNE, which proposed a new semi-supervised learning model. Combining the advantages of first-order and second-order estimation, SDNE can capture the global and local structural properties of the graph. The unsupervised part uses a deep auto-encoder to learn the second-order similarity, and the supervised part uses a Laplace feature map to capture the first-order similarity. Figure 2C illustrates the SDNE algorithm flow. By inputting the node embedding *S*_*i*_ in the model, where *S*_*i*_ is compressed by the auto-encoder, the feature is then reconstructed. Finally, its loss function is defined as follows:


(2) O2=Σ||S-′iSi||22

LINE is another method based on deep learning, which optimizes the first-order and second-order similarities (Figure 2D). The first-order similarity is used to describe the local similarity between pairs of nodes in the graph. The second-order similarity is described as two nodes in the graph not having directly connected edges, but there are common neighbor nodes, which indicate that the two nodes are similar.

#### Introduction to IC-Based Method

In this paper, we chose two typical IC-based methods to measure the semantic similarity of GO terms, based on Jiang and Conrath (1997) and Rel (Paul and Meeta, 2008). The IC of a term is inversely proportional to the frequency of the term being used to annotate genes in a given corpus, such as the UniProt database. The IC of a GO term *g* is defined by the negative log-likelihood and is given by


(3) IC(g)=-log⁡p(g)

(4) $$p\left(g\right)=\frac{freq\left(g\right)}{N}$$

where *p*(*g*) is the frequency of term *g* and its offspring in a specific GO annotated corpus. *N* represents the total number of annotated proteins in the corpus. If there are 50 annotated proteins in a corpus and 10 of them are annotated by term *g*, the annotation frequency of term *g* is *p*(*g*) = 0.2.

Jiang and Conrath and Rel’s methods rely on comparing the attributes of terms in GO. Jiang and Conrath’s method considered the fact that the semantic similarity between two terms is closely related to the nearest common ancestor corresponding to the two terms. The semantic similarity between two terms is estimated by calculating the amount of IC in the nearest common ancestor. Jiang and Conrath’s and Rel’s similarities are expressed as follows:


(5) $$sim_{J\&C}\left(g_{1},g_{2}\right)=2^{*}IC\left(g_{c}\right)-IC\left(g_{1}\right)-IC\left(g_{2}\right)$$

(6) $$sim_{Rel}\left(g_{1},g_{2}\right)=\frac{2^{*}IC\left(g_{c}\right)}{IC\left(g_{1}\right)+IC\left(g_{2}\right)}+\left(1-p\left(g_{c}\right)\right)$$

where *g*_*c*_ is the most informative common ancestor of *g*_1_ and *g*_2_ in the ontology. Given two proteins *P*_*m*_ and *P*_*n*_ annotated with GO terms *G*_*m*_ = {*g*_1_,⋯,*g*_*i*_} and $G_{n}=\left\{g_{1}^{{}^{\prime }},\cdots ,g_{j}^{{}^{\prime }}\right\}$, we used the Best Match Average (BMA) method to compute the similarity between two sets of GO terms, which can be expressed as follows:


(7) $$BMA(P_{m},P_{n})=\frac{1}{2}(\frac{1}{n}\sum_{g_{m}\in G_{m}}\underset{{}_{{g^{\prime }}_{n}\in G_{n}}}{\max}\;sim(g_{m},{g^{\prime }}_{n})+\frac{1}{m}\sum_{{g^{\prime }}_{n}\in G_{n}}\underset{g_{m}\in G_{m}}{\max}\;sim(g_{m},{g^{\prime }}_{n}))$$

where $sim\left(g_{m},g_{n}^{\prime }\right)$ is the similarity between term *g*_*m*_ and term *g*′_*n*_, which could have been calculated using IC-based similarity methods.

#### Protein Similarity Calculation

Each node in the GO graph is represented as a low-dimensional feature vector by considering the topology feature using a graph embedding method. Usually, a protein is annotated by several GO terms. For example, the protein “P03882” is annotated by the GO terms “GO:0004519,” “GO:0005739,” “GO:0006314,” and “GO:0006397.” Since a set of GO terms can be represented by its corresponding set of vectors, the similarity between proteins can be calculated based on the similarity of the two sets of GO vectors. Therefore, for any GO term *g*_*i*_, we use SDNE (Wang et al., 2016), DeepWalk (Perozzi et al., 2014), LINE (Tang et al., 2015), and Node2vec (Grover and Leskovec, 2016) graph embedding methods to learn the low-dimensional feature vector *v*_*i*_.

We let *G*_*m*_ = {*g*_1_,*g*_2_,⋯,*g*_*m*_} and $G_{n}=\left\{g_{1}^{\prime },g_{2}^{\prime },\cdots ,g_{n}^{\prime }\right\}$ denote the sets of GO terms that annotated proteins *P*_*m*_ and *P*_*n*_; thus, *V*_*m*_ = {*v*_1_,*v*_2_,⋯,*v*_*m*_} and $V_{n}=\left\{v_{1}^{\prime },v_{2}^{\prime },\cdots v_{n}^{\prime }\right\}$ denote the sets of vectors that correspond to *G*_*m*_ = {*g*_1_,*g*_2_,⋯,*g*_*m*_} and $G_{n}=\left\{g_{1}^{\prime },g_{2}^{\prime },\ldots g_{n}^{\prime }\right\}$, respectively. In this paper, we use the idea of DTW to calculate the similarity between two sets of vectors, which is denoted as DTW distance. The smaller the value, the more similar the two proteins. The GO embedding of the two proteins’ annotations is concatenated as *V*_*m*_ and *V*_*n*_, and the lengths are *m* and *n*, respectively (*m* ≠ *n*). For constructing the matrix *D*_*m×n*_, the element *D*(*v*_*m*_, *v*′_*n*_) represents the distance between points *v*_*m*_ and *v*′_*n*_ and can be expressed as follows:


(8) $$D\left(v_{m},v_{n}^{\prime }\right)=\min\left\{ \begin{array}{c} \begin{array}{c} D\left(v_{m-1},v_{n}^{\prime }\right)=Dist\left(v_{m-1},v_{n}^{\prime }\right)+d\left(v_{m},v_{n}^{\prime }\right) \end{array}\\ \begin{array}{c} D\left(v_{m},v_{n-1}^{\prime }\right)=Dist\left(v_{m},v_{n-1}^{\prime }\right)+d\left(v_{m},v_{n}^{\prime }\right)\\ D\left(v_{m-1},v_{n-1}^{\prime }\right)=Dist\left(v_{m-1},v_{n-1}^{\prime }\right)+2d\left(v_{m},v_{n}^{\prime }\right) \end{array} \end{array}\right.$$

We used the DTW distance method to find a path *W* through several lattice points in the matrix. The shortest path is the distance between the set of vectors *V*_*m*_ = {*v*_1_,*v*_2_,…*v*_*m*_} and $V_{n}=\left\{v_{1}^{\prime },v_{2}^{\prime },\ldots v_{n}^{\prime }\right\}$. We then calculated the distance used to measure the similarity between the two proteins. The process for calculating the DTW distance is presented in Supplementary Figure 1.

For any protein *P*_*i*_, the low-dimensional feature ω_*i*_ is directly learned from the GOA graph, which contains the information of term–term and term–protein relations. We use the cosine distance of the proteins’ vector ω to measure the similarity of the proteins. Cosine distance can be expressed as follows:


(9) $$D\left(P_{m},P_{n}\right)=\mathrm{cosine}\left(\omega_{m},\omega_{n}\right)=\frac{\omega_{m}\cdot \omega_{n}}{||\omega_{m}||||\omega_{n}||}$$

#### Link Prediction and Evaluation Metrics

When it is difficult to use a unified standard to measure the advantages and disadvantages of a network model, link prediction can be used as a unified comparison method for the similarity nodes in the network. It provides a standard to measure the reliability of the structure of the network. In the comprehensive evaluation, we use two commonly used evaluation indicators, AUC (Lobo, 2010) and AUCPR (Yu and Park, 2014), widely used in dichotomy. Therefore, to evaluate the available networks constructed based on different graph embedding methods in the GO graph and GOA graph, we perform link prediction experiments on the protein similarity network and evaluate the accuracy of the prediction results. For any undirected network *G*(*V*,*E*), we let *E* be the complete set of $C_{\left|V\right|}^{2}$ node pairs. We first remove 20% of the existing edges *E*_*r*_ in the network. The remaining 80% of the edges *E*_*s*_ are then divided into *E*_*p*_ and *E*_*t*_, where *E*_s_ = *E*_p_ ∪ *E*_*t*_, *E*_*P*_ ∩ *E*_t_ = ∅, and *E* = *E*_*r*_ ∪ *E*_*s*_. Given a link prediction method, each pair of unconnected node pairs *v*_*x*_ and *v*_*y*_ is given a link probability of two nodes. Sorting all the node pairs according to the score value in descending order, we have the top node pair with the highest link probability. The calculation process of the AUC value is presented in Supplementary Figure 2. The value of AUCPR is affected by the precision and recall value. For a link prediction experiment, accuracy is defined as the proportion of accurate prediction among the top *L* prediction edges. If *m* prediction edges exist, sort the link probability score value in descending order. If *m* of the top *L* edges are in the *E*_*t*_, the precision is defined as follows:


(10) $$Precision=\frac{m}{L}$$

The number of existing edges in the network *M* = *E*−*E*_*r*_, where *m* is the number of edges predicted by the prediction algorithm. The recall index is defined as follows:


(11) $$\mathrm{Recall}=\frac{m}{M}$$

The similarity between nodes is an essential precondition for link prediction, and the more similar the two nodes are, the more likely that a link exists between them. The similarity of network-based structural information definition is called structural similarity. Link prediction accuracy based on structure similarity depends on whether the structure similarity can grasp target structure characteristics. In the link prediction task, there are many methods to calculate the structural similarity between nodes, such as the following:

##### Common neighbors index

Common Neighbors (CN) (Li et al., 2018) similarity can be called structural equivalence, that is, if two nodes have multiple common neighbors, they are similar. In the link prediction experiment, CN index basic assumption is that if two unconnected nodes have more common neighbors, they are more likely to be connected. For nodes *v*_*x*_ and *v*_*y*_ in the protein similarity network, their neighbors are defined as Γ(*x*) and Γ(*y*), and the similarity of the two nodes is defined as the number of their CN. The index of CN is defined as follows:


(12) $$S_{xy}=\left|\Gamma \left(x\right)\cap \Gamma \left(y\right)\right|=\left(A^{2}\right)$$

where *S* represents the similarity matrix and *A* represents the adjacency matrix of the graph. CN index is based on local information similarity index.

##### Jaccard index

Based on the common neighbors and considering the influence of the node degree at both ends, the Jaccard (JC) similarity index (Ran et al., 2015) is proposed. JC not only considers the number of two nodes’ common neighbors but also considers the number of all their neighbors. JC is defined as follows:


(13) $$S_{xy}=\frac{\left|\Gamma \left(x\right)\cap \Gamma \left(y\right)\right|}{\left|\Gamma \left(x\right)\cup \Gamma \left(y\right)\right|}=\frac{{\left(A^{2}\right)}_{xy}}{||\Gamma \left(x\right)\cap \Gamma \left(y\right)||}$$

##### Resource allocation index

Resource Allocation (RA) (Dianati et al., 2005) index considers the attribute information of the common neighbors of two nodes. In the link prediction process, the common neighbor nodes with higher degrees play a lesser role than those with lower degrees, and the weight of the common neighbor nodes decreases in the form of 1k. An example is presented in Supplementary Figure 3. RA index (Dianati et al., 2005) is defined as follows:


(14) $$S_{xy}=\sum_{z\in \Gamma \left(x\right)\cap \Gamma \left(y\right)}\frac{1}{K_{\text{z}}}$$

where *K*_*z*_ is the degree of the common neighbors of nodes *v*_*x*_ and *v*_*y*_. The calculation process of the RA similarity index is shown in Supplementary Figure 3. Assuming that each node’s resources are distributed equally to its neighbors, the RA index calculates a node’s received resources, which is the similarity between nodes *v*_*x*_ and *v*_*y*_.

## Results

### Comparison of Protein Similarity and the Actual PPI Network Coincidence Degree

We downloaded the human yeast protein interaction network from the String database. We then mapped the proteins to the UniProt database, filtered out those proteins that could not be found in the UniProt database, and removed duplicate edges. After filtering, the Yeast dataset consisted of 2,877 proteins with 228,468 interactions, and the Human dataset consisted of 6,882 proteins with 892,054 interactions. Finally, to verify the validity of our calculated protein similarity network, we compared protein similarity and the actual PPI network coincidence degree.

**FIGURE 3:**
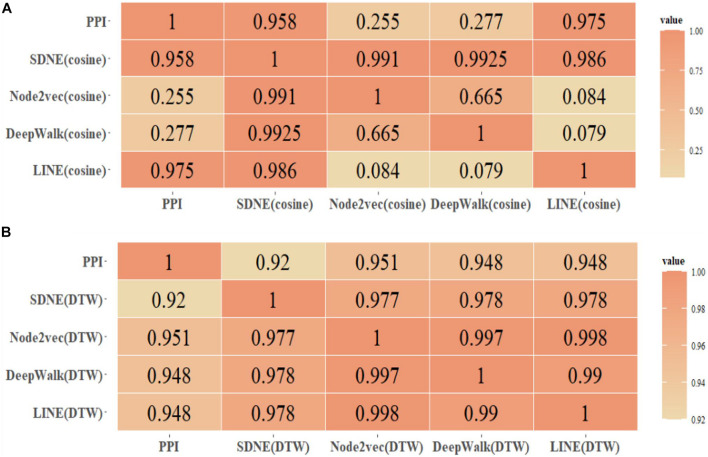
Human protein similarity network (τ > 0.4) and PPI coincidence degree. **(A)** Cosine, **(B)** DTW.

This paper only shows the Human dataset experiment results in Figure 3, and the Yeast dataset results are shown in Supplementary Figures 4, 5.

We selected the protein similarity networks (τ > 0.4) and compared them with the PPI dataset downloaded from the String database to analyze the coincidence degree of the Human and Yeast protein networks. Furthermore, we compared the edge coincidence of the protein similarity network based on different graph embedding methods (as shown in Figure 3). The calculation was based on $\frac{E_{a}\cap E_{b}}{E_{a}}\left(E_{a}>E_{b}\right)$.

By comparing the GO(DTW) and GOA(cosine) methods, it can be seen that the Node2vec graph embedding method performed best in the GO graph. SDNE and LINE methods performed better in the GOA graph, and there was little difference between them in the GOA graph and GO graph. However, Node2vec and DeepWalk performed better in the GO graph. In general, the performance of protein similarity calculation based on different graph embedding methods in the GO graph was better than in the GOA graph. As shown, using graph embedding methods can be effective in calculating protein similarity in GO and GOA graphs. We also proved that using the DTW method to calculate different dimensional protein vector similarities is feasible.

### Comparison of Link Prediction Results Based on Different Graph Embedding Methods in GO Graph

**FIGURE 4:**
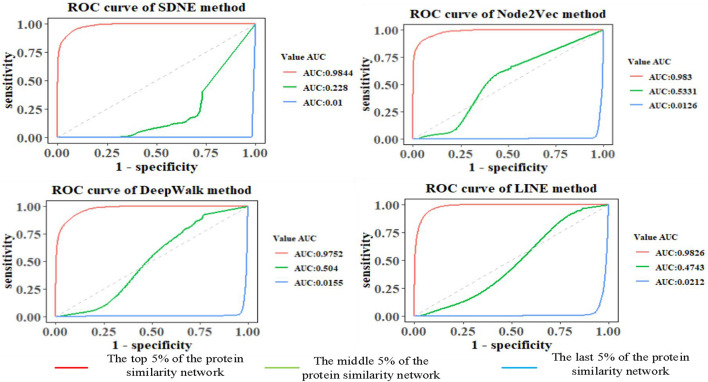
Comparison of prediction results of Human protein similarity networks.

The features of GO terms are learned from the GO graph based on different graph embedding methods, and the similarity among proteins is calculated. By selecting the top 5%, middle 5%, and the last 5% of the protein similarity network data, the link prediction is computed for the filtered protein similarity network, and the AUC and AUCPR values are calculated (as shown in Figure 4 and Table 2). This paper only shows the Human dataset experiment result, and the Yeast dataset result is shown in Supplementary Figure 6 and Supplementary Table 1.

**TABLE 2:** AUCPR value of protein similarity prediction in the Human dataset.

Method	The top 5% of the network	The middle 5% of the network	The last 5% of the network
SDNE	0.9105	0.0076	0.0052
Node2vec	**0.9115**	**0.0143**	**0.0055**
DeepWalk	0.8220	0.0127	0.0052
LINE	0.7117	0.0097	0.0052

*Bold means the best result in the comparative experiment.*

We can see that as the similarity of network nodes decreases, the value of AUC decreases. In the top 5% of the protein similarity network, the proteins are more similar, but for AUCPR values, we can see that the performance of the Node2vec method is the best in all the top, middle, and the last 5% of the protein similarity networks. The Node2vec method introduces BFS and DFS into the generation process of the random walk sequence by introducing two parameters *p* and *q*. BFS focuses on the adjacent nodes and characterizes a relatively local graph representation; that is, the BFS can explore the local structural properties of the graph, while the DFS can explore the global similarity in context. We found that the AUC value of protein similarity calculated by the graph embedding method decreased gradually with the decrease in the value of the screening protein similarity. Furthermore, it is shown that the edge connection of the protein similarity network calculated by the graph embedding method is reliable.

We also found that the Node2vec graph embedding method performed well in calculating the Yeast protein similarity network (as shown in Supplementary Figure 6 and Supplementary Table 1). Therefore, the GO term vectors fused the local and global information of nodes in the GO graph and contain more information, so the GO(DTW) method performs better in computing protein similarity.

### Comparison of Link Prediction Results Based on Different Graph Embedding Methods in the GOA Graph

**FIGURE 5:**
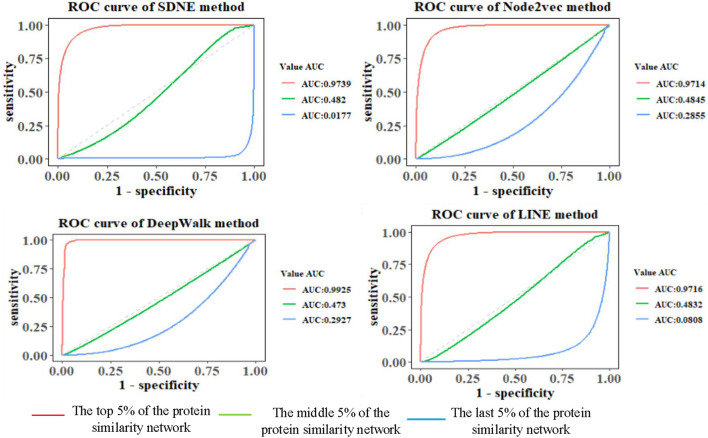
Comparison of prediction results of Human protein similarity networks.

To reflect the influence of the structure information of the GO annotation on proteins, the features of proteins are learned from the GOA graph based on different graph embedding methods, and the similarity among proteins is calculated (as shown in Figure 5 and Table 3). This paper only shows the Human dataset experiment result, and the Yeast dataset result is presented in Supplementary Figure 7 and Supplementary Table 2.

**TABLE 3:** AUCPR value of Human protein similarity prediction.

Method	The top 5% of the network	The middle 5% of the network	The last 5% of the network
SDNE	0.6578	0.0100	0.0052
Node2vec	**0.8758**	**0.0105**	**0.0069**
DeepWalk	0.8719	0.0094	0.0053
LINE	0.8189	0.0095	0.0053

*Bold means the best result in the comparative experiment.*

We screened the top, middle, and last 5% of the protein similar networks and performed the link prediction experiments to observe the values of AUC and AUCPR under different methods. The AUC and AUCPR values decreased gradually with the decrease in the percentage selected. Therefore, it can be seen that the performance of the Node2vec method in the GOA(cosine) method is also better than other graph embedding methods. For the Yeast protein similarity network, we also performed the same experiment and obtained the same experimental conclusions as described above. We found that SDNE graph embedding methods also showed excellent performance in the Yeast dataset (as shown in Supplementary Table 2). This is because the SDNE method also defines first-order and second-order similarities. Therefore, calculating the protein similarity network based on these vectors achieved excellent results in the prediction task.

### Comparison of Link Prediction Results of Protein Similarity Calculated by IC-Based Method and Based on Graph Embedding Methods

We studied the application of different graph embedding methods to calculate protein similarity in GO and GOA graphs. We screened the top 5% of the protein similarity networks for link prediction analysis (as shown in Table 4). Furthermore, we performed an experiment that calculated the density of the protein similarity network based on graph embedding and IC-based methods (as shown in Table 5). This paper only presents the Human dataset experiment results, and the Yeast dataset result is presented in Supplementary Tables 3, 4.

**TABLE 4:** AUCPR and AUC values of Human protein similarity prediction (the top 5% of the similarity network).

Method	AUC	AUCPR
SDNE (cosine/DTW)	0.9699/**0.9739**	0.9015/**0.9105**
Node2vec (cosine/DTW)	0.9714/**0.983**	0.8758/**0.9115**
DeepWalk (cosine/DTW)	**0.9925**/0.9752	**0.8719**/0.8220
LINE (cosine/DTW)	**0.9839**/0.9716	**0.8189**/0.7117
Rel.	0.9067	0.1519
Jiang and Conrath	0.8409	0.0669

*Bold means the best result in the comparative experiment.*

**TABLE 5:** Comparison of Human protein similarity network density between different methods.

Method	Nodes	Edges	Density
SDNE (cosine/DTW)	4,797/2,024	1,183,801/713,961	0.1/**0.3**
Node2vec (cosine/DTW)	6,882/2,807	2,841,303/1,183,762	0.12/**0.3**
DeepWalk (cosine/DTW)	6,882/3,079	1,183,876/1,183,707	0.05/**0.2**
LINE (cosine/DTW)	5,586/1,660	1,183,815/206,650	0.07/**0.15**
Rel	5,902	870,987	0.05
Jiang and Conrath	5,883	870,986	0.05

*Bold means the best result in the comparative experiment.*

The link prediction results from these methods are compared as follows. From Table 4, it can be seen that the similarity calculation of proteins based on different graph embedding methods is superior to that of the IC-based methods. We also performed the above experiment for Yeast datasets, and the same conclusion was obtained (as shown in Supplementary Table 3). It can be seen that the SDNE and Node2vec graph embedding methods show good performance in the GO graph. Analyzing the density of the top 5% of the human protein similarity networks, it can be seen that the density of the protein similarity network calculated by the graph embedding method is higher than that calculated by IC-based methods. Therefore, it is shown that the protein similarity network calculated by the IC-based method is sparse, and the similarity of proteins is not as high as that calculated by the graph embedding method. Thus, in the IC-based method, the AUCPR value obtained in link prediction is lower. We also verified this conclusion on the Yeast dataset (as shown in Supplementary Table 4).

Based on different graph embedding methods, the features of the GO terms were learned into the vector representations through fusing the topology of the GO graph. Thus, we could capture the global information based on the graph embedding method, and its learned vectors could calculate the similarity between proteins by the DTW distance similarity. As can be seen from the results of the link prediction, the GO(DTW) method performed better than GOA(cosine), and most of the protein similarity networks calculated by the GO(DTW) method are denser than those calculated by the GOA(cosine) method.

### Similarity Indexes’ Results

We performed three different link prediction similarity index experiments on the top 5% of the protein similarity network and found that based on different similarity indexes, the difference in the AUC value is small, which indicates that the calculated protein similarity network structure has improved (as shown in Table 6). This paper only presents the Human dataset experiment result, and the Yeast dataset result is presented in Supplementary Table 5.

**TABLE 6:** Prediction results under different similarity indexes (the top 5% of the Human protein similarity network).

Similarity index	CN	JC	RA
SDNE (cosine/DTW)	0.9694/0.981	0.9739/0.9843	**0.9818**/**0.9886**
Node2vec (cosine/DTW)	0.9598/0.9809	0.9714/0.9843	**0.9856**/**0.9886**
DeepWalk (cosine/DTW)	0.9772/0.981	0.9856/0.9842	**0.9885**/**0.9884**
LINE (cosine/DTW)	0.9703/0.9716	0.9716/0.9825	**0.9874**/**0.9853**

*Bold means the best result in the comparative experiment.*

Among the three different similarity evaluation indexes, we found that the AUC value of the RA similarity index based on link prediction is slightly higher than the other two similarity indexes. Furthermore, the results showed that the top 5% of the protein similarity network had higher AUC values in different similarity indexes of link prediction, indicating that the graph embedding method effectively calculated protein similarity. We obtained the same conclusion in the experiment with the Yeast dataset (as shown in Supplementary Table 5).

## Discussion

Gene Ontology is one of the many biological ontology languages. Its emergence and development reduce the confusion of biological concepts and terms, provide a three-layer (BP, MF, and CC) structure of system definition, and describe the functions of proteins. Therefore, it is important to understand protein function based on GO terms to describe protein similarity.

In this paper, by fusing the GO terms’ topology information, we learned the features of GO terms and proteins into vector representations in GO and GOA graph based on different graph embedding methods. Then, the similarity of proteins was calculated based on these vectors using DTW and cosine similarity. Finally, protein similarity networks were screened by selecting different percentages, and a link prediction experiment was used to evaluate the prediction accuracy of different networks. The experimental results indicate that the graph embedding method is better than the IC-based method in protein similarity calculation. Among the two graph embedding methods, the performance of the GO(DTW) method is better than that of the GOA(cosine) method. This is because the GO terms and proteins are treated equally in the GOA graph, and some information may be ignored when learning protein low-dimensional embedding. Therefore, the coincidence degree between the protein similarity network calculated by the GOA(cosine) method and the actual PPI data is not as high as that calculated by the GO(DTW) method. There are potential limitations to our method. First, we transformed directed graphs into undirected graphs, which might result in a loss of structural information. We also treated the GO terms and the proteins equally in the GOA graph, which may ignore some information. Therefore, in our future study, we plan to learn the protein representations in the graph by combining the information in the directed graph and by considering representation learning of heterogeneous graphs that contain GO terms and proteins.

## Data Availability Statement

The original contributions presented in the study are included in the article/Supplementary Material, further inquiries can be directed to the corresponding author/s.

## Author Contributions

YZ conceived the idea and prepared the experimental data. ZW and YZ debugged the code, conducted the experiments, interpreted the results, and wrote and edited the manuscript. SW and JS advised the study and reviewed the manuscript. All authors contributed to the article and approved the submitted version.

## Conflict of Interest

The authors declare that the research was conducted in the absence of any commercial or financial relationships that could be construed as a potential conflict of interest.

## Publisher’s Note

All claims expressed in this article are solely those of the authors and do not necessarily represent those of their affiliated organizations, or those of the publisher, the editors and the reviewers. Any product that may be evaluated in this article, or claim that may be made by its manufacturer, is not guaranteed or endorsed by the publisher.

## Footnotes

1
http://geneontology.org/page/download-ontology

2
http://www.ebi.ac.uk/GOA
