# Supplementary figures and images for: Comparative Analysis of Unsupervised Protein Similarity Prediction Based on Graph Embedding
Source: Front Genet. 2021 Sep 22;12:744334. doi: 10.3389/fgene.2021.744334 (PMC8493040; doi:10.3389/fgene.2021.744334)

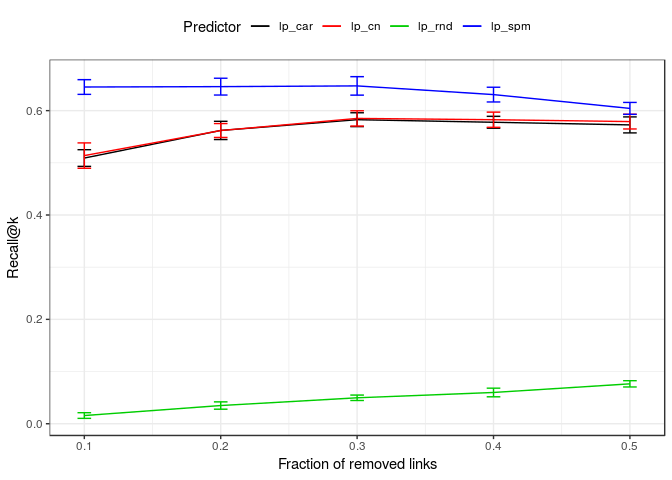

Supplement: Supplementary file 2 [file Data_Sheet_2.ZIP › code/LinkPrediction-master/README_files/figure-markdown_github/unnamed-chunk-5-1.png]

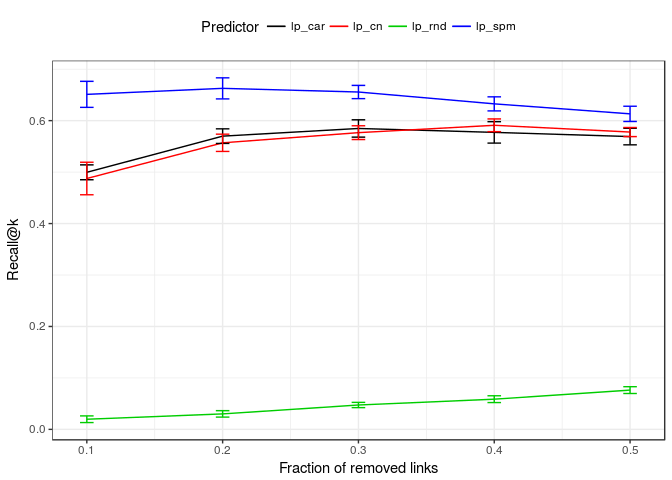

Supplement: Supplementary file 2 [file Data_Sheet_2.ZIP › code/LinkPrediction-master/README_files/figure-markdown_github-ascii_identifiers/unnamed-chunk-5-1.png]

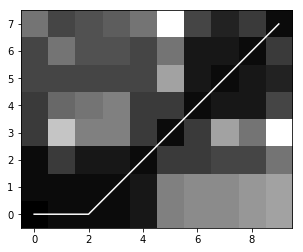

Supplement: Supplementary file 2 [file Data_Sheet_2.ZIP › code/dtw-master/acc.png]
