# Supplementary material for: Comparative Analysis of Unsupervised Protein Similarity Prediction Based on Graph Embedding
Source: Front Genet. 2021 Sep 22;12:744334. doi: 10.3389/fgene.2021.744334 (PMC8493040; doi:10.3389/fgene.2021.744334)
Supplement: Supplementary file 3 [file Data_Sheet_3.docx]

Figure S1 | The framework of DTW method.

Figure S2 | The example of AUC calculation.

Figure S3 | An example of RA similarity metrics.


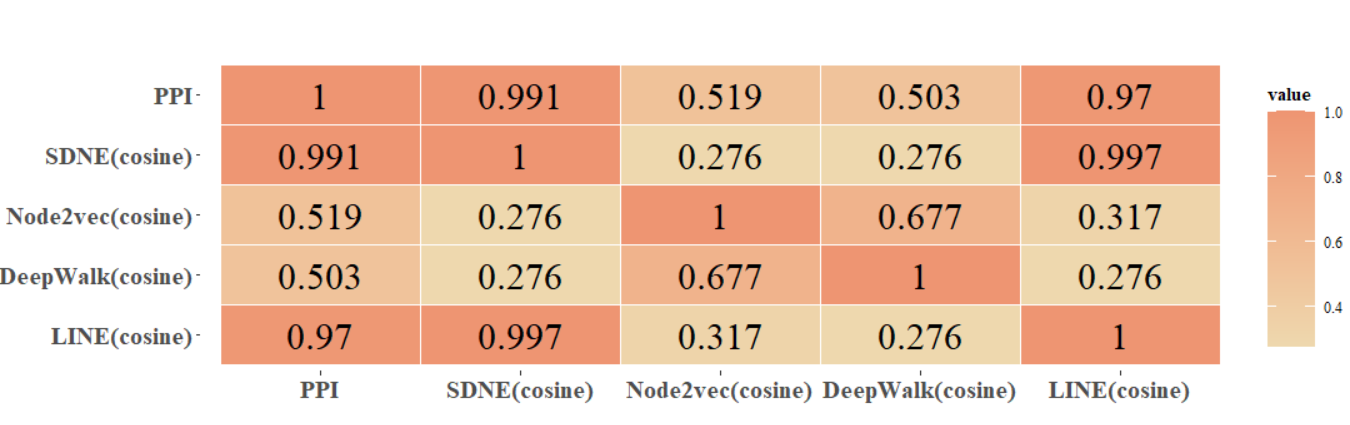


Figure S4 | Yeast protein similarity network (cosine) ($\tau>0.4$) and PPI coincidence degree.


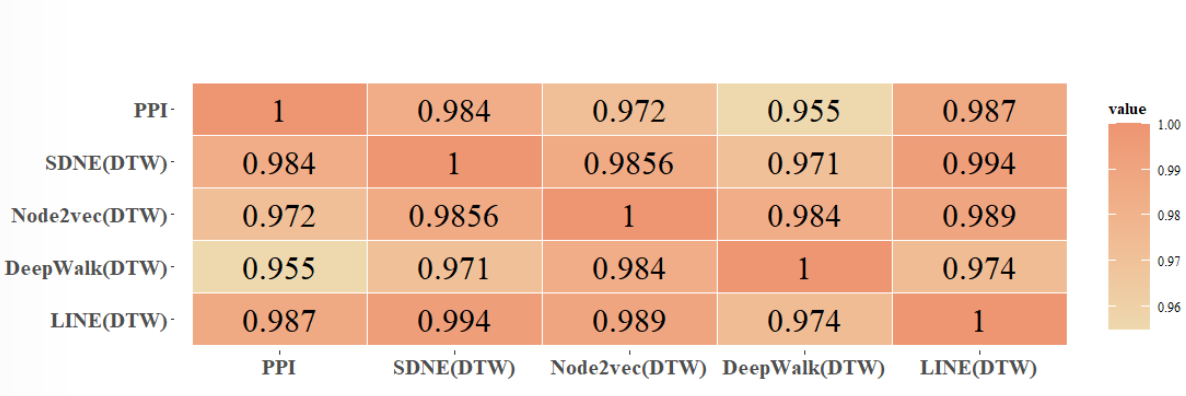


Figure S5 | Yeast protein similarity network (DTW) ($\tau>0.4$) and PPI coincidence degree.

Figure S6 | Comparison of prediction results of Yeast protein similar networks.

Table S1 | AUCPR value of protein similar prediction on Yeast.

| Method | The top 5% of networks | The middle 5% of network | The last 5% of network |
| --- | --- | --- | --- |
| SDNE | 0.8739 | 0.0173 | 0.0052 |
| Node2vec | **0.8758** | **0.0223** | **0.0056** |
| DeepWalk | 0.8719 | 0.0108 | 0.0052 |
| LINE | 0.8130 | 0.0094 | 0.0052 |

Figure S7 | Comparison of prediction results of Yeast protein similar networks.

Table S2 | AUCPR value of yeast protein similarity prediction.

| Method | The top 5% of network | The middle 5% of network | The last 5% of network |
| --- | --- | --- | --- |
| SDNE | **0.7094** | **0.0098** | **0.0083** |
| Node2vec | 0.5472 | 0.0097 | 0.0081 |
| DeepWalk | 0.6936 | 0.0094 | 0.0052 |
| LINE | 0.5256 | 0.0097 | 0.0057 |

Table S3 | AUCPR and AUC values of yeast protein similarity prediction (The top 5% similarity network).

| Method | AUC | AUCPR |
| --- | --- | --- |
| SDNE(cosine/DTW) | 0.9845/0.9699 | 0.7094/**0.8739** |
| Node2vec(cosine/DTW) | 0.9782/0.9814 | 0.5472/**0.8758** |
| DeepWalk(cosine/DTW) | 0.9841/0.9855 | 0.6936/**0.8719** |
| LINE(cosine/DTW) | 0.9669/0.9839 | 0.5472/**0.8130** |
| Rel. | 0.9643 | 0.4136 |
| Jiang&Cornth | 0.9421 | 0.2425 |

Table S4 | Comparison of Yeast protein similarity network density between different methods.

| Method | Nodes | Edges | Density |
| --- | --- | --- | --- |
| SDNE(cosine/DTW) | 2332/777 | 206716/206716 | 0.07/**0.42** |
| Node2vec(cosine/DTW) | 2876/1382 | 1183876/206570 | **0.28**/0.21 |
| DeepWalk(cosine/DTW) | 2877/2332 | 1183876/206716 | **0.28**/0.07 |
| LINE(cosine/DTW) | 2374/1660 | 206817/206650 | 0.07/**0.15** |
| Rel(cosine/DTW) | 2503 | 156938 | 0.05 |
| Jiang&Cornth(cosine/DTW) | 2505 | 156938 | 0.05 |

Table S5 | Prediction results under different similarity indexes (The top 5% Yeast protein similarity network).

| Similarity index | CN | JC | RA |
| --- | --- | --- | --- |
| SDNE(cosine/DTW) | 0.974/0.9665 | 0.9845/0.9699 | **0.9877**/**0.9788** |
| Node2vec(cosine/DTW) | 0.9668/0.9838 | 0.9782/0.9838 | **0.9832**/**0.9888** |
| DeepWalk(cosine/DTW) | 0.9829/0.9901 | 0.9894/0.9841 | **0.9929**/**0.9922** |
| LINE(cosine/DTW) | 0.9675/0.979 | 0.9669/0.9839 | **0.9786**/**0.9869** |
